# Lung cancer combined with diffuse peritoneal and mesenteric amyloidosis detected on ^18^F-FDG PET/CT: A case report

**DOI:** 10.1097/MD.0000000000025961

**Published:** 2021-05-28

**Authors:** JianJie Wang, Bin Zhao, Tianbin Song, Jidong Sun

**Affiliations:** a Department of Nuclear Medicine, Shougang Hospital of Peking University, Shijingshan District; b Department of Radiology and Nuclear Medicine, Xuanwu Hospital of Capital Medical University, Xicheng District; c Department of Neurosurgery, Aviation General Hospital of China Medical University, Chaoyang District, Beijing, People's Republic of China.

**Keywords:** ^18^F-fluorodeoxyglucose positron emission tomography/computed tomography, amyloidosis, case report, pulmonary neoplasms

## Abstract

**Rationale:**

Amyloidosis is a heterogeneous group of diseases characterized by extracellular deposition of amyloid fibrils. Lung carcinoma is rarely reported to be associated with AA amyloidosis. With regard to the manifestation of amyloidosis infiltrating organs, most of the cases focus on the heart, liver, kidneys, and peripheral nervous system. Amyloidosis with diffuse abdominal involvement in combination with pulmonary squamous cell carcinoma carcinoma is an exceptionally rare occurrence.

**Patient concerns:**

A 70-year-old man was admitted to hospital for a 2-month history of repeated cough, low grade fever, hemoptysis and left back shoulder pain, which was not relieved by nonsteroid anti-inflammatory drugs. Meanwhile, he complained of intermittent diffuse abdominal discomfort and chronic persistent constipation.

**Diagnoses:**

The patient was diagnosed with poorly differentiated lung squamous cell carcinoma and diffuse peritoneal and mesenteric amyloidosis based on the pathological biopsy.

**Interventions:**

The patient received surgery and chemotherapy for lung tumor. He did not receive any treatment against amyloidosis.

**Outcomes:**

The patient died of a severe respiratory infection.

**Lessons:**

This case indicates that lung carcinoma is suspected to play a causative role in the development of amyloidosis. In addition, amyloidosis should be considered in the differential diagnosis in cases in which diffuse greater omentum, peritoneal, and mesenteric calcifications on ^18^F-2-fluoro-2-deoxy-D-glucose(^18^F-FDG) photon emission computed tomography (PET/CT).

## 1 Introduction

Secondary (AA) amyloidosis is defined as extracellular deposition of fibrils that are composed of serum amyloid A (SAA) protein,^[1]^ which is produced predominantly by hepatocytes in response to signaling from pro-inflammatory cytokines.^[2]^ Chronic inflammatory disease is a major cause of AA amyloidosis; however, AA amyloidosis can also occur synchronously or asynchronously as a result of neoplastic processes.^[1,3–6]^ Systemic AA amyloidosis in lung carcinoma is extremely rare in the literature.^[7–9]^ To the best of our knowledge, there has only been 1 reported case, that of a pulmonary squamous cell carcinoma patient who received treatment with chemoradiation and an immune checkpoint inhibitor and subsequently presented with systemic AA amyloid deposition.^[8]^ In addition, amyloidosis involved in the peritoneal and mesenteric regions is rare, with the most common sites of organ involvement including the heart, liver, kidneys, and peripheral nervous system.^[10]^ Here, we report a case of pathological proven abdominal amyloidosis with concurrent primary lung squamous cell carcinoma.

## 2 Case presentation

A 70-year-old man was admitted to hospital for a 2-month history of repeated cough, low grade fever, hemoptysis, and left back shoulder pain, which was not relieved by nonsteroid anti-inflammatory drugs. Meanwhile, he complained of intermittent diffuse abdominal discomfort and chronic persistent constipation. He had a history of a traffic accident 2 years ago, and his chest wall was wounded, although the thoracic computed tomography (CT) scanning at that moment did not present any significant bone fracture. Incidentally, a 2.4 × 2.5-cm solid nodule was detected in the left upper lung. He was an active smoker with a 200-pack-year smoking history. His body temperature was 36.6 °C, and blood pressure was 130/90 mm Hg. Physical examination showed slightly decreased breath sounds in left apical area on auscultation, with left back pain when pressing the scapular area; other physical examination parameters were unremarkable. Laboratory tests showed that his blood count, serum electrolytes, and renal and liver functions were within normal limits. SAA protein was 34 mg/L (normal 0–10 mg/L). Laboratory test showed elevated tumor markers, with CEA of 5.9 ng/ml (normal 0–5 ng/ml) and cytokeratin fragment of 6.9 ng/ml (normal 0.1–3.3 ng/ml). Serum immunoelectrophoresis revealed 2 minor M-peaks in the gamma region, without significant monoclonal gammopathy, and the concentration of 2 M-peaks was 1.4 g/dl. Urine protein electrophoresis were negative for Bence-Jones, whereas the rest of the hematological and biochemical parameters were all normal.

**Figure 1:**
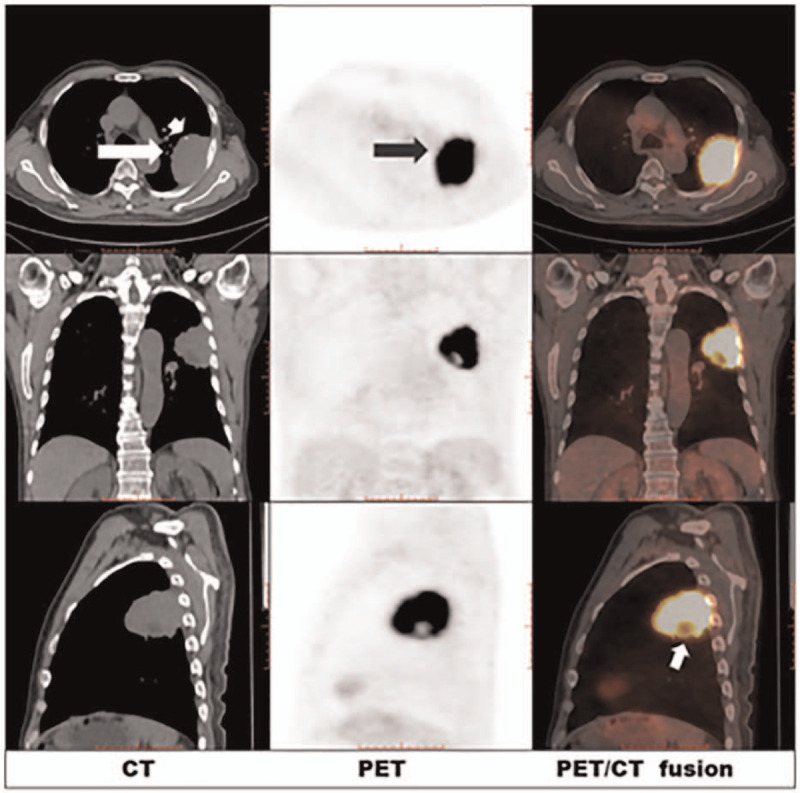
The CT images of the thoracic demonstrates a 5.7 × 7.9 cm lobulated solid mass with burr-like margins in the left upper lobe of lung (white arrow), with marginal punctate calcifications was noted (arrowhead). PET images reveal that the mass in left lung presenting intense FDG uptake (black arrow), with the SUV_max_ of 19.9. PET/CT fusion images the short white arrow indicate a local decreased FDG radioactivity in the lower margin of the lesion. SUV_max_ = the maximum of standard uptake value.

**Figure 2:**
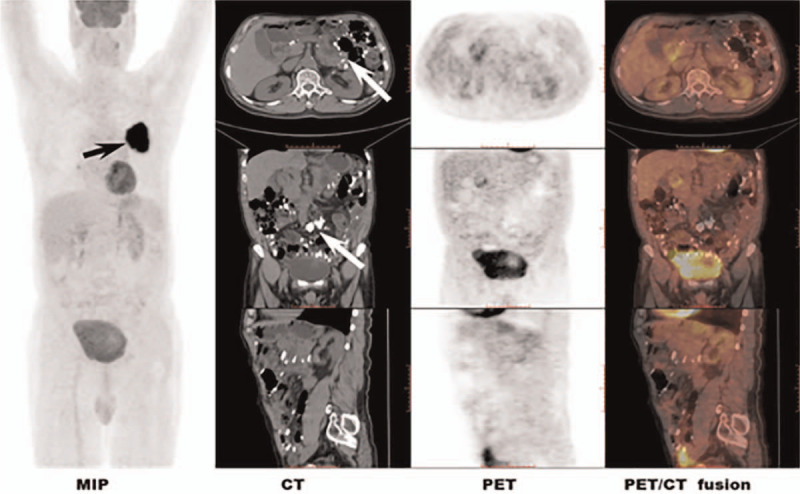
The MIP image demonstrates an irregular mass in the left upper lung field, exhibiting intense FDG uptake (black arrow). The abdominal CT reveal diffuse, amorphous calcifications in the greater omentum, mesentery of small bowel and colon (white arrow), with peritoneum thickening moderately. There is no apparent FDG-avidity in the corresponding PET images, with SUVmax of 1.0. SUV_max_ = the maximum of standard uptake value.

The ^18^F-2-fluoro-2-deoxy-D-glucose (^18^F-FDG) positron emission tomography/computed tomography (PET/CT) MIP revealed a large mass with intense FDG uptake in the left upper lung lobe (the maximum of standard uptake value =19.9, Fig. 1) and diffuse amorphous calcifications located in the greater omentum, mesentery of small bowel and colon (Fig. 2).

**Figure 3:**
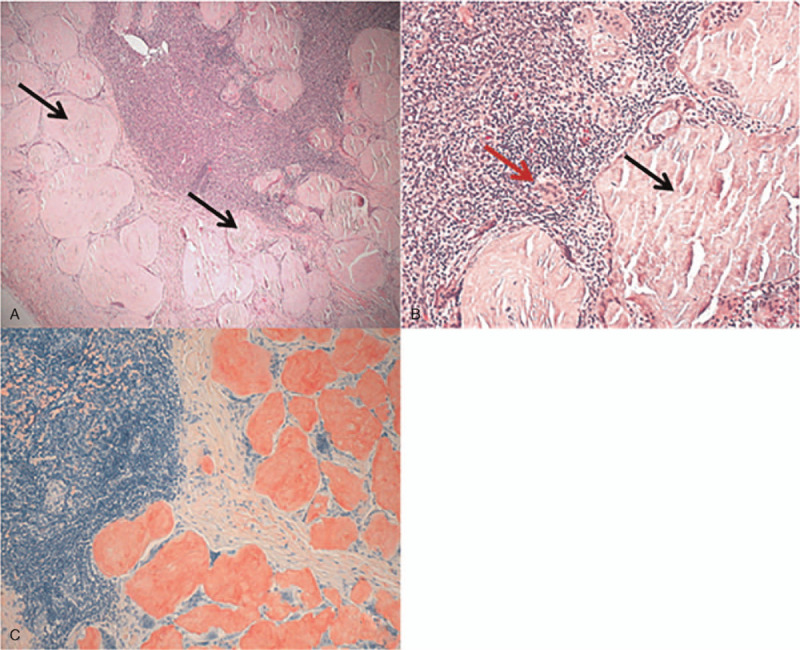
H & E stain of the resected peritoneum specimens reveal the amorphous depositions of amyloid in the lymph node tissue, surrounded by numerous ploykaryocytes and lymphocytes, and plasma cells surrounding amyloid material (A, ×40, B, ×100). Immunohistochemical finding of the depositions of amyloid lymphatic tissue shows that Congo red staining is positive (C, ×100).

A percutaneous CT-guided thoracoscopic biopsy of the lung mass was subsequently performed, showing poorly differentiated squamous cell carcinoma. Meanwhile, pathological findings showed that the specimens of resected peritoneum contained amorphous, homogeneous material with some polyclonal plasma cells, lymphocytes and giant cells, while additional stain with Congo red revealed characteristic amyloid depositions (Fig. 3).

The patient underwent surgery to remove the lung cancer, followed by chemotherapy. He did not receive any treatment against amyloidosis. Unfortunately, his condition rapidly deteriorated at the beginning of the fourth cycle of chemotherapy, and he died of a severe respiratory infection.

## 3 Discussion

Amyloidosis consists of a heterogeneous group of disorders characterized by widespread extracellular deposition of amyloids in multiple organs and tissues, which can present in unusual ways and with a wide range of clinical features.^[10–12]^ Based on immunohistochemistry, there are 4 major categories of amyloidosis: primary or immunoglobulin light chain (AL) disease, hereditary or mutant transthyretin disease, secondary or amyloid protein A disease, and dialysis-associated or β-2 microglobulin disease.^[13,14]^

AA amyloidosis is well known to develop in the setting of persistently high levels of SAA, which is an acute-phase reactant produced predominantly by hepatocytes, but also by macrophages, endothelial cells, and smooth muscle cells, and regulated by proinflammatory cytokines.^[15]^ AA amyloidosis is associated with diseases such as chronic infections, aging, chronic inflammatory diseases, Hodgkin disease and solid tumors.^[16]^ The incidence of systemic AA amyloidosis in cancer patients is 0.1% to 0.4%.^[9]^ Among solid tumors, hypernephroma, and renal cell carcinoma are the most associated with AA amyloidosis, and at autopsy, 2% to 3% of patients with renal carcinoma are found to have amyloids.^[8,9]^ Only 1 case of amyloidosis was detected in a series of 4033 lung cancer cases.^[17]^

Reports of amyloidosis and lung cancer are rare. Gueutin et al^[9]^ reported a case of glomeruli and kidney vessel walls AA amyloidosis caused by stage IIIB lung adenocarcinoma. The patient underwent palliative treatment for renal function, but deteriorated rapidly. Nobata et al^[2]^ reported the first case of systemic AA amyloidosis due to a solitary lung metastasis from completely resected moderately differentiated renal cell carcinoma. A further case of gastrointestinal AA amyloidosis in a patient with poorly differentiated pulmonary squamous cell carcinoma treated with chemoradiation and immune checkpoint inhibitors has also been described,^[7]^ in which the patient was confirmed to have profuse hemoptysis or hematemesis and died shortly after admission. Barceló et al^[8]^ presented 1 case of an association between poorly differentiated epidermoid lung cancer and rectal mucosa AA amyloidosis and reviewed the literature. It seemed that the mechanisms of amyloid deposits within the tumor are different from systemic amyloidosis.^[18,19]^ Our patient also succumbed to disease quickly before there was a chance to treat amyloidosis. One reason for the low incidence of malignancy-related systemic AA amyloidosis is the short-term nature of SAA elevation.^[2]^

In view of the small number of reported cases, the correlation between the pathological type of lung cancer and amyloidosis is unknown. High serum concentrations of SAA have been found in association with the dissemination of lung cancer.^[20–22]^ In our case, elevated SAA was noticed. Barceló et al^[8]^ believe that serum circulating precursors of amyloid fibrils may be more significant than expected and could be the biological mechanism responsible for the development of systemic amyloidosis in patients with lung cancer. Nobata et al^[2]^ found AA amyloid deposited in vascular and perivascular areas, but SAA was not produced by tumor cells in the present study. Based on several possible mechanisms that malignant cells could directly produce SAA, and antitumor lymphocytes, macrophages, or malignant cells could secrete pro-inflammatory cytokines. These cells may therefore be important for the induction of SAA production and development of AA amyloidosis. The precise mechanisms underlying amyloid formation in association with solid tumors is unknown.

Several case reports have described the role of ^18^F-FDG PET/CT in AL amyloidosis. Andor et al^[23]^ showed that increased FDG uptake was not found in any affected organ containing amyloid in 10 patients with systemic amyloidosis. Joo et al^[24]^ showed that the maximum of standard uptake value was significantly increased in 15 of 22 organs in patients with primary systemic AL amyloidosis (68.2%; 10 hearts, 2 kidneys, 1 colon, 1 ileum, and 1 liver). In our case, the absence of bowel wall infiltration may account for the absence of FDG activity.

Gastrointestinal tract involvement occurs in only 8% of patients with systemic AL amyloidosis, mostly involving the small bowel.^[25]^ Diffuse mesenteric and retroperitoneal fat-infiltration amyloidosis is rarely reported.^[26,27]^ In our case, abdominal CT revealed predominant diffuse amorphous calcifications along the greater omentum, mesentery of small bowel, and colon, with slightly peritoneal thickening, lacking apparent infiltrating mass, which exhibits some difference from the previous studies. The extensive calcifications within the mesenteric and peritoneal soft tissue infiltration lesions restrict the differential diagnosis. Peritoneal mesothelioma may present on CT with peritoneal nodules or masses with ascites.^[28,29]^ Calcified metastasis along peritoneal surfaces can frequently result from ovarian carcinoma, and serous cystadenocarcinomas contain histologic calcifications in approximately 30% of cases.^[30,31]^ Chronic tuberculosis or granulomatous disease should be considered in the differential diagnosis in cases of multiple calcified lymph nodes.^[32,33]^

Systemic AA amyloidosis is still a life-threatening complication of chronic inflammatory diseases. Despite the success of anti-inflammatory treatment, the prognosis of some AA patients is poor.^[34]^ Treatment for AA amyloidosis is targeted at decreasing SAA production. Monoclonal antibodies against TNF-α and IL-6, which are newer treatments for chronic autoimmune disorders, have been shown to be effective in the management of AA amyloidosis.^[15]^ Nevertheless, novel therapeutic strategies targeting the formation of amyloid fibrils and amyloid deposition may generate new expectations for patients with AA amyloidosis.^[9]^

## 4 Conclusion

Our case report highlights that AA amyloidosis is rarely associated with lung squamous cell carcinoma and emphasizes that amyloidosis should be considered in the differential diagnosis in cases in which diffuse amorphous calcifications over the greater omentum, mesentery of the small bowel, and colon with thickening peritoneum are detected on FDG PET/CT imaging.

## Acknowledgments

We would like to thank Editage (www.editage.com) for English language editing.

## Author contributions

**Conceptualization:** Jianjie Wang.

**Writing – original draft:** Jianjie Wang, Bin Zhao, Jidong Sun.

**Writing – review & editing:** Tianbin Song.
